# TIGIT^+^ CD4^+^ T Cell Enrichment in Colorectal Liver Metastases Is Associated with Shorter Survival

**DOI:** 10.3390/cancers18182977

**Published:** 2026-09-15

**Authors:** Janusz von Renesse, Jakob Langsdorf, Elisabeth Kalb, Carolin Beer, David Digomann, Loreen Natusch Bufe, Daniela E. Aust, Jürgen Weitz, Lena Seifert, Adrian M. Seifert

**Affiliations:** 1 Department of Visceral, Thoracic and Vascular Surgery, University Hospital Carl Gustav Carus, TU Dresden University of Technology, 01307 Dresden, Germany; janusz.vonrenesse@ukdd.de (J.v.R.);; 2 National Center for Tumor Diseases (NCT), University Hospital Carl Gustav Carus, TU Dresden University of Technology, 01307 Dresden, Germany; 3 German Cancer Consortium (DKTK), Partner Site Dresden, German Cancer Research Center (DKFZ), 69120 Heidelberg, Germany; 4 Faculty of Medicine University Hospital Carl Gustav Carus, TU Dresden University of Technology, 01307 Dresden, Germany; 5 Institute of Pathology and Tumor and Normal Tissue Bank of the University Cancer Center (UCC), Faculty of Medicine Carl Gustav Carus, University Hospital Carl Gustav Carus, TU Dresden University of Technology, 01307 Dresden, Germany; 6 Else Kröner Clinician Scientist Professor for Translational Tumor Immunological Research, 01307 Dresden, Germany

**Keywords:** colorectal cancer liver metastases, tumor-infiltrating lymphocytes, T cells, TIGIT, PD-1

## Abstract

Background/Objectives: The immune microenvironment of colorectal cancer liver metastases (CRCLM) differs from that of primary colorectal tumors and may influence responses to immunotherapy. We aimed to characterize T cell subsets and checkpoint receptor expression in CRCLM and explore associations with overall survival. Methods: We performed flow cytometric profiling of matched peripheral blood, non-tumor liver tissue, and CRCLM specimens from 17 patients undergoing hepatic metastasectomy. A separate cohort of 19 patients was studied using ex vivo expanded TILs. Kaplan–Meier/log-rank analyses were complemented by Cox models using dichotomized and continuous marker values, median-cut-off and permutation sensitivity analyses, and post hoc clinical covariate comparisons. Results: CRCLM showed relative enrichment of CD4^+^ T cells and depletion of CD8^+^ T cells. In the fresh cohort, 16 patients (12 deaths) were evaluable for CRCLM TIGIT^+^ CD4^+^ cells. The optimized split was associated with shorter survival for the high group (HR 5.26, 95% CI 1.54–17.94; log-rank *p* = 0.0036). The median-cut-off analysis showed the same direction (HR 3.17, 95% CI 0.92–11.00; *p* = 0.0551), as did the continuous model (HR 1.69 per 10-percentage-point increase, 95% CI 0.82–3.45; *p* = 0.153). In the expanded cohort, the optimized high-versus-low HR was 17.47 (95% CI 3.23–94.59; *p* < 0.001), and the continuous HR was directionally concordant (HR 2.68, 95% CI 0.96–7.51; *p* = 0.060). Clinical characteristics were balanced between optimized low and high groups. Conclusions: TIGIT^+^ CD4^+^ T cell enrichment showed an exploratory association with shorter survival in CRCLM and represents a promising candidate for further prognostic and functional investigation. The separate expanded cohort provided complementary evidence supporting this observation.

## Simple Summary

Colorectal cancer frequently spreads to the liver, where the local immune environment may limit the effectiveness of immunotherapy. We examined immune cells from blood, non-tumor liver tissue, and colorectal cancer liver metastases. Freshly isolated immune cells from 17 patients were analyzed, and a separate cohort of 19 patients was studied after ex vivo expansion of tumor-infiltrating lymphocytes. Higher TIGIT expression on CRCLM-infiltrating CD4^+^ T cells was associated with shorter survival in optimized cut-off analyses, whereas PD-1 expression was not. Continuous Cox estimates showed the same direction of association, and a similar survival pattern was observed in the separate expanded-cell cohort. Together, these findings identify TIGIT^+^ CD4^+^ T cell enrichment as a promising candidate marker in CRCLM and provide a rationale for prospective biomarker and functional investigation.

## 1. Introduction

In modern multimodal cancer therapy, the discovery and advancement of checkpoint inhibitors have marked a significant breakthrough. However, despite these advancements, a substantial proportion of tumors do not respond to current immunotherapies [1]. This lack of response is often attributed to insufficient T cell infiltration, T cell exhaustion, and an immunosuppressive tumor microenvironment [2]. In colorectal carcinoma (CRC) the use of checkpoint inhibitors is mostly effective in microsatellite instability-high (MSI-H) tumors [3]. Colorectal cancer liver metastases (CRCLM) are the predominant cause of cancer-related mortality in patients with CRC. The liver represents a tolerogenic immune niche characterized by antigen presentation under tolerizing conditions, which may promote T cell dysfunction and limit anti-tumor immunity [4]. These differences in the microenvironments compared to the primary tumor pose a unique challenge in developing effective immunotherapies and may reduce their efficacy through the difference in the targeted immune cell subset composition [5,6]. TIGIT (T cell immunoreceptor with Ig and ITIM domains) is a member of the immune checkpoint family and a promising candidate for novel immunotherapies [7]. Despite strong preclinical rationale, recent clinical trials targeting TIGIT have shown mixed results, highlighting the need to identify tumor-specific immune contexts in which TIGIT signaling is biologically relevant [8]. TIGIT is predominantly expressed by T and NK cells. TIGIT expressed by tumor-infiltrating lymphocytes (TILs) interacts with its ligands CD155 (PVR) and CD112 (nectin-2), CD113 (nectin-3), and PVRL4 (nectin-4), which can be upregulated on tumor cells, attenuating proliferation and cytotoxicity through co-signaling [9]. Interestingly, the role and distribution of TIGIT in CRCLM have been poorly studied despite high expression on CD8^+^ T cells in CRC [10,11]. TIGIT competes with the co-stimulatory receptor CD226 for binding to CD155, thereby suppressing T cell activation and cytotoxic function [12]. Notably, high expression of CD226 is associated with improved survival in CD8^+^ T cells in CRCLM [13]. Recently, the role of CD4^+^ T cells in the tumor microenvironment has gained increased attention [14]. Besides their classical function as helper cells and the immunosuppressive effects of regulatory T cells (Tregs), a cytotoxic role for CD4^+^ T cells has also been demonstrated [15,16,17]. Tailoring immune checkpoint blockade (ICB) to the specific immune infiltrate of a tumor may enhance treatment efficacy while reducing cost and adverse effects. Consequently, detailed characterization of TILs should be integrated with diagnostic tools [18]. However, limited tissue availability, often due to small tumor size and the requirement for histopathological evaluation, can restrict direct flow cytometric analysis [6]. To address this issue, T cells can be expanded ex vivo from small tumor fragments, providing sufficient material for phenotypic and functional assessments [19,20]. Nevertheless, ex vivo expansion can alter checkpoint expression, and the extent to which expanded cells resemble the in situ immune landscape remains unclear. To explore the immune landscape of CRCLM, we performed flow cytometric analysis of T cell subsets and investigated their expression of TIGIT and PD-1 in matched blood, liver tissue, and CRCLM from 36 patients with metastatic CRC. Freshly isolated T cells from 17 CRCLM patients were assessed for exploratory associations with overall survival. Ex vivo expanded T cells from a separate cohort of 19 patients were analyzed to assess feasibility and cohort-level similarities in checkpoint patterns using a less tissue-intensive approach.

## 2. Materials and Methods

### 2.1. Patients

A total of 36 patients undergoing resection of CRCLM at the University Hospital Dresden between January 2019 and April 2021 were included in this study. In 17 patients, T cells were immediately isolated from fresh CRCLM tissue and adjacent non-tumor liver. In 19 patients, T cells were expanded ex vivo from resected tumor tissue. Blood samples were collected immediately prior to the surgical resection of the metastases. Fresh tumor and adjacent non-tumor liver were collected from the resected specimen by a trained pathologist. The study was conducted in accordance with the principles of the Declaration of Helsinki. The study protocol was approved by the Ethics Committee of the Technical University of Dresden (EK446112017). Written consent for participation in the study was obtained from all patients. The clinical characteristics of the patients are summarized in Table 1.

### 2.2. Flow Cytometry

Peripheral blood mononuclear cells (PBMCs) were isolated from the blood through density centrifugation. This involved mixing the blood in a 1:1 ratio with PBS and layering it over a Pancoll solution (PAN-biotech, Aidenbach, Germany). Centrifugation was performed for 25 min at 800× *g* at 18 °C. The tissue provided by the pathologist was manually minced using surgical scissors. Subsequently, the tissue chunks were digested for 30 min at 37 °C in an enzyme mix containing Collagenase Type IV (Sigma Aldrich, St. Louis, MO, USA), Hyaluronidase from bovine testes (Sigma Aldrich), and DNase I from bovine pancreas (Sigma Aldrich) in DMEM (GIBCO, Thermo Fisher Scientific, Waltham, MA, USA). The digested tissue was then filtered through a 100 µm mesh. The suspension was first centrifuged at 50 g for 3 min to remove hepatocytes and cellular debris, and the supernatant was used for further analyses. To isolate the immune cell fraction, CD45-magnetic beads (Miltenyi Biotec, Bergisch Gladbach, Germany) were employed according to the manufacturer’s protocol. Subsequently, the single-cell suspension was first incubated with an Fc-block, stained with a LIVE/DEAD marker, and then stained with an antibody mix for flow cytometry. The samples were analyzed using a FACS Fortessa flow cytometer (BD Biosciences, San Jose, CA, USA), and the data were analyzed using FlowJo v10.7.1.

A common sequential gating strategy was applied to freshly isolated and ex vivo expanded samples. Lymphocytes were selected by FSC-A/SSC-A, followed by live-cell, singlet (FSC-H/FSC-A), and CD45^+^ leukocyte gates. After CD45 selection, CD4/CD8 quadrant gating identified CD4^+^ single-positive (Q1) and CD8^+^ single-positive (Q3) cells, which were analyzed as the CD4^+^ and CD8^+^ populations, respectively; double-positive and double-negative events were excluded from both denominators. PD-1 and TIGIT positivity was quantified within the respective single-positive population. Representative checkpoint and isotype-control plots are shown in Supplementary Figure S1.

### 2.3. RNA Expression Data Analysis

The publicly accessible dataset GSE207194, which includes bulk RNA sequencing data from a total of 60 samples, comprising 36 CRCLMs and 24 primary CRCs paired with the CRCLM, was used [13]. The data were obtained from the Gene Expression Omnibus (GEO) database. For our analyses, we used normalized FPKM (Fragments Per Kilobase of transcript per Million mapped reads) data. To calculate gene signatures for the exhausted T cell score (comprising *HAVCR2*, *LAG3*, *PDCD1*, *CXCL13*, and *LAYN*), we employed the geometric mean of the log2 (1^+^ gene expression matrix) transformation. For visualization and calculation of correlations, we used the R package “rempysc” version 0.1.7.

### 2.4. T Cell Ex Vivo Expansion

Initially, liver and CRCLM tissue were cut into 1 mm3 pieces and then cultured in RPMI-1640 medium (Gibco, Thermo Fisher Scientific, Waltham, MA, USA), supplemented with 10% heat-inactivated human serum (AB, male), 1% Glutamax (Gibco), 1% Penicillin/Streptomycin (Gibco), and 200 IU/mL IL-2. Once visible T cell growth was observed after a period of one to two weeks, the medium was filtered to obtain a single-cell suspension. This suspension was then stained for flow cytometry in the same manner as the freshly isolated immune cells. In parallel, paired isolated PBMCs were treated under the same incubation conditions. Subsequently, the ex vivo expanded TILs were analyzed together with the treated PBMCs.

### 2.5. Statistical Analysis

A two-sided Wilcoxon rank-sum (Mann–Whitney U) test was used to compare TIGIT gene expression between primary CRC and CRCLM. To compare cell populations and marker expression among blood, liver tissue, and CRCLM, a repeated-measures ANOVA was performed. In cases where values were missing in a particular group because of low cell numbers, a mixed-effects analysis was used. Overall survival was defined as time from surgery to death or last follow-up; patients alive at last follow-up were censored. Kaplan–Meier curves were compared using two-sided log-rank tests. Kaplan–Meier figures display censor marks, log-rank *p* values, and numbers at risk; confidence bands were omitted for visual clarity. Univariable Cox proportional hazards models were used to estimate HRs with 95% CIs for high versus low expression and for each 10-percentage-point increase in continuous marker expression. Proportional hazards were assessed using scaled Schoenfeld residuals; a quadratic marker term was evaluated as a simple test of departure from log-linearity. For optimized cut-off analyses, all observed marker values were screened while requiring at least one quarter of the full merged cohort in each group; the split with the smallest log-rank *p* value was selected. Median cut-offs were analyzed as a sensitivity analysis. Four tumor checkpoint comparisons (PD-1 and TIGIT in CD4^+^ and CD8^+^ T cell subsets) were evaluated within each cohort and cut-off method. No multiplicity adjustment was prespecified; exact unadjusted *p* values are reported with post hoc Benjamini–Hochberg and Bonferroni adjustments and a 10,000-permutation test that repeats the cut-off search after permuting marker values. Post hoc high-versus-low clinical comparisons used Wilcoxon rank-sum tests for age and Fisher exact tests for categorical variables. Cox models adjusted the TIGIT^+^ CD4^+^ association for history of systemic therapy before liver surgery. Analyses were performed using R version 4.6.0 and GraphPad Prism 9.3.1. Two-sided *p* values below 0.05 were considered nominally significant. Percentages of checkpoint-positive cells in CRCLM samples from the fresh and expanded cohorts were compared using two-sided Mann–Whitney U tests. *p* values were adjusted for the four prespecified comparisons of PD-1 and TIGIT expression in CD4^+^ and CD8^+^ cells using the Holm method.

## 3. Results

### 3.1. Reversal of the CD4^+^/CD8^+^ T Cell Ratio Reveals CD4^+^ T Cell Predominance in CRCLM

**Figure 1:**
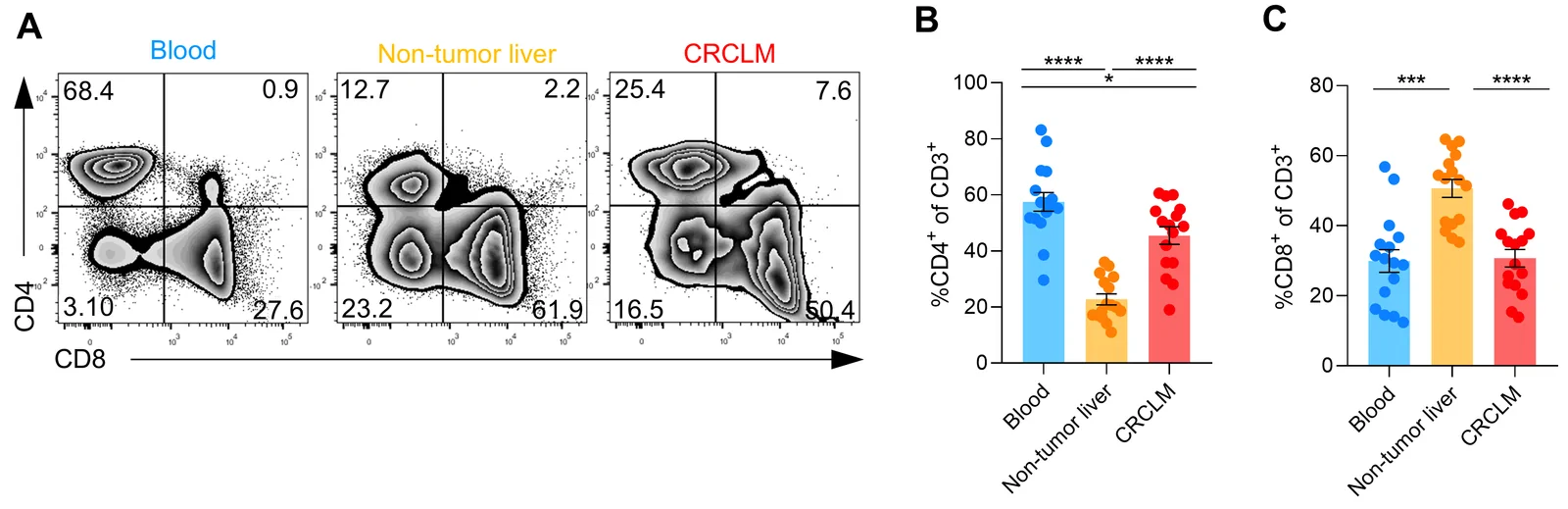
Reversal of the CD4^+^ /CD8^+^ T cell ratio reveals CD4^+^ T cell predominance in CRCLM. (**A**) Representative flow cytometry plots of CD4^+^ and CD8^+^ T cell subsets from matched blood, non-tumor liver tissue, and CRCLM specimens of patients with colorectal cancer liver metastases. (**B**,**C**) Quantitative analysis of the percentages of (**B**) CD4^+^ and (**C**) CD8^+^ cells within the CD3^+^ T cell population in matched blood, non-tumor liver tissue, and CRCLM specimens. Each dot represents an individual patient. Repeated-measures one-way ANOVA was performed to compare the different specimen types. *, *p* < 0.05; ***, *p* < 0.001; ****, *p* < 0.0001.

To characterize the T cell composition in CRCLM, we performed flow cytometry on matched samples of peripheral blood, non-tumor liver tissue, and CRCLM lesions (n = 17). CD4^+^ T cells were the predominant T cell subset in peripheral blood, but their proportion significantly decreased within the non-tumor liver tissue and CRCLM while strongly increasing within CRCLM compared to the non-tumor liver tissue (Figure 1A,B). CD8^+^ T cells showed a reversed distribution being most abundant in non-tumor liver tissue, resulting in a reduced CD4^+^ to CD8^+^ T cell ratio in the non-tumor liver (Figure 1A,C). Notably, this ratio was again inverted within CRCLM lesions: CD4^+^ T cells were markedly increased (Figure 1B), while CD8^+^ T cells were reduced (Figure 1C), indicating a shift toward CD4^+^ T cell predominance within the tumor microenvironment. To corroborate these findings and assess whether this CD4^+^ T cell enrichment was unique to liver metastases or already present in primary colorectal tumors, we analyzed publicly available bulk RNA sequencing data from paired primary CRC and CRCLM. CD4 and CD8 expression levels were both elevated in CRCLM compared to primary CRC, with a more pronounced increase in CD4, highlighting the CD4 dominance (Supplementary Figure S2A,B).

### 3.2. PD-1 Expression Increases in CD4^+^ T Cells Within CRCLM but Is Not Associated with Patient Survival

**Figure 2:**
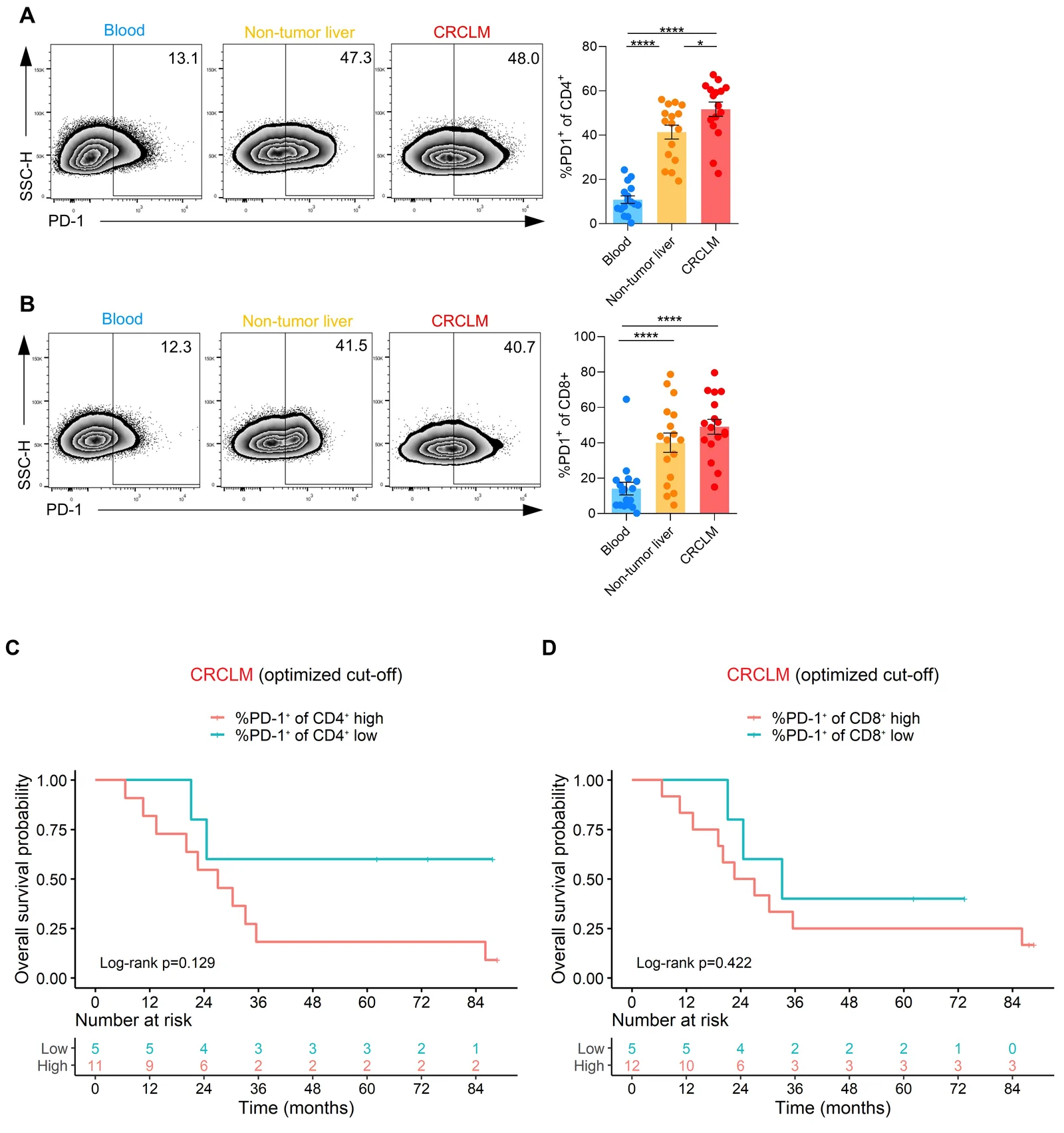
PD-1 expression increases in CD4^+^ T cells within CRCLM but is not associated with patient survival. (**A**,**B**) Representative flow cytometry plots illustrating PD-1 expression (**left** panels) on (**A**) CD4^+^ T cells and (**B**) CD8^+^ T cells, accompanied by quantitative dot plots of the PD-1 positive percentage of these cell populations from matched patient blood, non-tumor liver tissue, and CRCLM specimens (**right** panels). (**C**) Kaplan–Meier survival analysis comparing patients stratified by high and low PD-1 expression on CRCLM-infiltrating CD4^+^ T cells, grouped by the optimized cut-off value. (**D**) Kaplan–Meier survival analysis comparing patients stratified by high and low PD-1 expression on CRCLM-infiltrating CD8^+^ T cells, grouped by the optimized cut-off value. A repeated-measures one-way ANOVA was performed to compare the different specimen types. *, *p* < 0.05; ****, *p* < 0.0001. *p*-values from the log-rank test are indicated within the respective Kaplan–Meier plots. Kaplan–Meier panels show censor marks, log-rank *p* values, and numbers at risk; confidence bands are omitted for visual clarity. For panel C, 16 patients and 12 deaths were analyzed: low, n = 5 with 2 deaths; high, n = 11 with 10 deaths; HR for high versus low 3.07 (95% CI 0.67–14.10). For panel D, 17 patients and 13 deaths were analyzed: low, n = 5 with 3 deaths; high, n = 12 with 10 deaths; HR 1.70 (95% CI 0.46–6.31).

To characterize the exhaustion profile of the T cell infiltrate in CRCLM, we analyzed the expression of the inhibitory receptor PD-1 by flow cytometry in matched blood, non-tumor liver, and CRCLM tissue samples. We observed that the non-tumor liver microenvironment alone was sufficient to induce a significant increase in PD-1 expression in both CD4^+^ and CD8^+^ T cells compared to blood (Figure 2A,B). Notably, a significant increase in PD-1^+^ CD4^+^ T cells was observed in CRCLM compared with non-tumor liver (Figure 2A), whereas PD-1 expression on CD8^+^ T cells did not show a significant increase in tumor tissue (Figure 2B). Because PD-1 has been associated with outcome in other malignancies, we explored its association with patient survival. Using the Kaplan–Meier survival analysis with optimized cut-offs identified with a best-fit model, we compared patients with high and low PD-1 expression on CD4^+^ and CD8^+^ T cells within CRCLM. However, no significant differences in overall survival were observed for either subset (Figure 2C,D). We further analyzed PD-1 expression in blood and non-tumor liver across both CD4^+^ and CD8^+^ T cells (Supplementary Figure S3A,B). Again, no significant survival differences were detected in any compartment. This remained unchanged when applying a median-based cut-off (Supplementary Figure S3C,D), indicating no detectable association between PD-1 expression alone and outcome in this cohort.

### 3.3. TIGIT^+^ CD4^+^ T Cell Enrichment in CRCLM Shows an Exploratory Association with Shorter Survival

**Figure 3:**
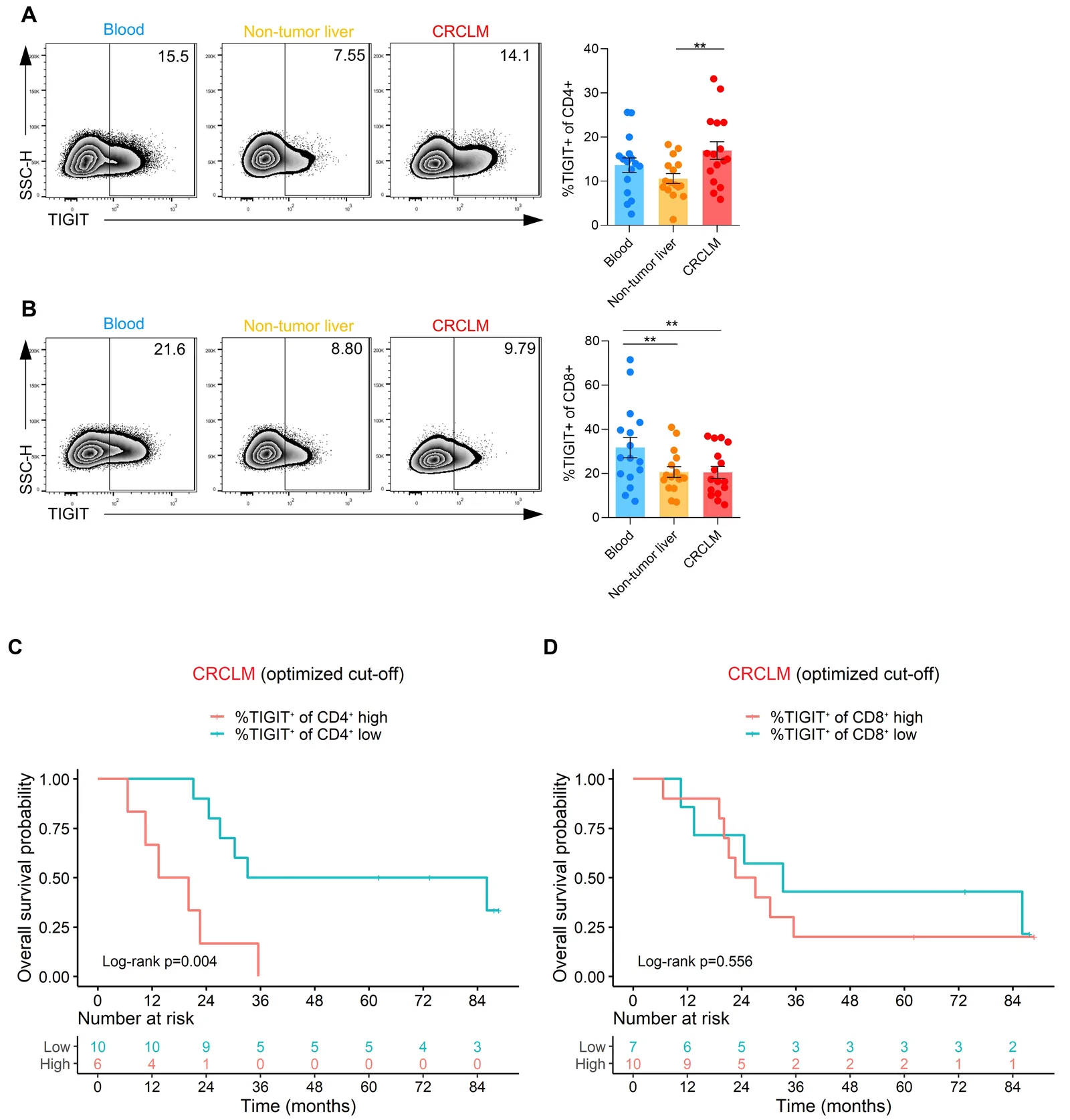
TIGIT^+^ CD4^+^ T cell enrichment in CRCLM shows an exploratory association with shorter survival. (**A**,**B**) Representative flow cytometry plots illustrating TIGIT expression (**left** panels) on (**A**) CD4^+^ T cells and (**B**) CD8^+^ T cells, accompanied by quantitative dot plots of the TIGIT positive percentage of these cell populations from matched patient blood, non-tumor liver tissue, and CRCLM specimens (**right** panels). (**C**) Kaplan–Meier survival analysis comparing patients stratified by TIGIT high and low expression levels of CRCLM-infiltrating CD4^+^ T cells, grouped by the optimized cut-off value. (**D**) Kaplan–Meier survival analysis comparing patients stratified by TIGIT high and low expression levels of CRCLM-infiltrating CD8^+^ T cells, grouped by the optimized cut-off value. A repeated-measures one-way ANOVA was performed to compare the different specimen types. **, *p* < 0.01. *p*-values from the log-rank test are indicated within the respective Kaplan–Meier plots. Kaplan–Meier panels show censor marks, log-rank *p* values, and numbers at risk; confidence bands are omitted for visual clarity, while 95% CIs are reported here and in the Results. For panel C, 16 patients and 12 deaths were analyzed: low, n = 10 with 6 deaths; high, n = 6 with 6 deaths; HR for high versus low 5.26 (95% CI 1.54–17.94). For panel D, 17 patients and 13 deaths were analyzed: low, n = 7 with 5 deaths; high, n = 10 with 8 deaths; HR 1.40 (95% CI 0.45–4.35).

To explore the expression profile of TIGIT in colorectal cancer and its liver metastases, we first analyzed publicly available bulk RNA sequencing data from paired primary CRC and CRCLM. We compared TIGIT expression levels between primary tumors and matched liver metastases, revealing that TIGIT transcript levels were significantly upregulated in CRCLM compared to primary CRC (Supplementary Figure S4A). Additionally, we observed a strong correlation between TIGIT expression and exhausted T cell gene signatures, both in primary CRC (Supplementary Figure S4B, left) and in CRCLM (Supplementary Figure S4B, right). These findings are consistent with increased TIGIT-associated regulation in the liver metastatic microenvironment. To validate these transcriptomic findings, we assessed TIGIT expression on tumor-infiltrating T cells by flow cytometry across matched peripheral blood, non-tumor liver, and CRCLM samples. While TIGIT expression on CD4^+^ T cells did not significantly differ between blood and non-tumor liver, we observed a marked increase in TIGIT^+^ CD4^+^ T cells in CRCLM compared to liver tissue (Figure 3A). In contrast, TIGIT-expressing CD8^+^ T cells were significantly decreased in both liver and tumor tissue compared to blood (Figure 3B). These results highlight a selective enrichment of TIGIT^+^ CD4^+^ T cells in CRCLM, whereas CD8^+^ T cells appear to have less TIGIT expression in both non-tumor liver and CRCLM tissue. Given this differential pattern, we explored associations with overall survival. For CRCLM-infiltrating TIGIT^+^ CD4^+^ T cells, 16 patients with 12 deaths were evaluable; one of the 17 fresh cohort patients lacked this marker value. The optimized cut-off was 16.3%, yielding 10 low patients (six deaths) and six high patients (six deaths). High expression was associated with shorter survival (HR 5.26, 95% CI 1.54–17.94; log-rank *p* = 0.0036; Figure 3C). With a median cut-off of 15.55%, the estimate was attenuated and not nominally significant (HR 3.17, 95% CI 0.92–11.00; log-rank *p* = 0.0551; Supplementary Figure S4E). A post hoc 10,000-permutation analysis accounting for selection of the optimal cut-off yielded *p* = 0.0339; after Benjamini–Hochberg adjustment across the four tumor checkpoint comparisons, q = 0.1356. TIGIT expression on CD8^+^ T cells was not associated with survival (optimized cut-off 13.6%; HR 1.40, 95% CI 0.45–4.35; 13 deaths/17 patients; log-rank *p* = 0.556; low, n = 7 with five deaths; high, n = 10 with eight deaths; Figure 3D). No survival differences were detected for TIGIT^+^ CD4^+^ or CD8^+^ cells in blood or non-tumor liver. In a continuous Cox model, each 10-percentage-point increase in CRCLM TIGIT^+^ CD4^+^ frequency corresponded to HR 1.69 (95% CI 0.82–3.45; *p* = 0.153). The proportional hazard and quadratic-term tests did not indicate departure from a simple linear, time-constant model (*p* = 0.538 and *p* = 0.677, respectively). The direction of association was concordant with the dichotomized analysis, supporting further investigation of CRCLM-infiltrating TIGIT^+^ CD4^+^ cells for prospective prognostic and functional investigation. Terminal T cell exhaustion, which is driven by chronic antigenic stimulation, is often marked by co-expression of multiple inhibitory checkpoint receptors. Recently, we reported a correlation between PD-1 and TIGIT expression in pancreatic cancer, suggesting coordinated checkpoint regulation [21]. We therefore assessed whether TIGIT expression correlated with PD-1 expression in matched blood, liver, and CRCLM tissues using flow cytometric data. In CD8^+^ T cells, we found a strong correlation between TIGIT and PD-1 expression across compartments (Supplementary Figure S5A). Specifically, TIGIT expression on CD8^+^ T cells in CRCLM correlated well with TIGIT levels in both blood and non-tumor liver (Supplementary Figure S5A). Furthermore, TIGIT expression on CD8^+^ T cells in CRCLM was significantly correlated with PD-1 expression on CD8^+^ T cells in the same tissue, supporting the notion of co-regulation and co-expression of exhaustion markers in this subset. In contrast, the correlations for CD4^+^ T cells were notably weaker. TIGIT expression on CD4^+^ T cells in CRCLM correlated significantly only with TIGIT expression in non-tumor liver tissue, but not with TIGIT expression in blood or PD-1 expression in CRCLM (Supplementary Figure S5A). These findings suggest that TIGIT^+^ CD4^+^ T cells in CRCLM are not strongly associated with classical exhaustion phenotypes, such as PD-1 co-expression. Taken together, these results indicate that TIGIT and PD-1 co-expression reflects canonical exhaustion predominantly in CD8^+^ T cells, whereas the TIGIT^+^ CD4^+^ T cell population in CRCLM may reflect distinct biology.

### 3.4. Ex Vivo Expanded TILs Provide a Complementary Cohort-Level Comparison

**Figure 4:**
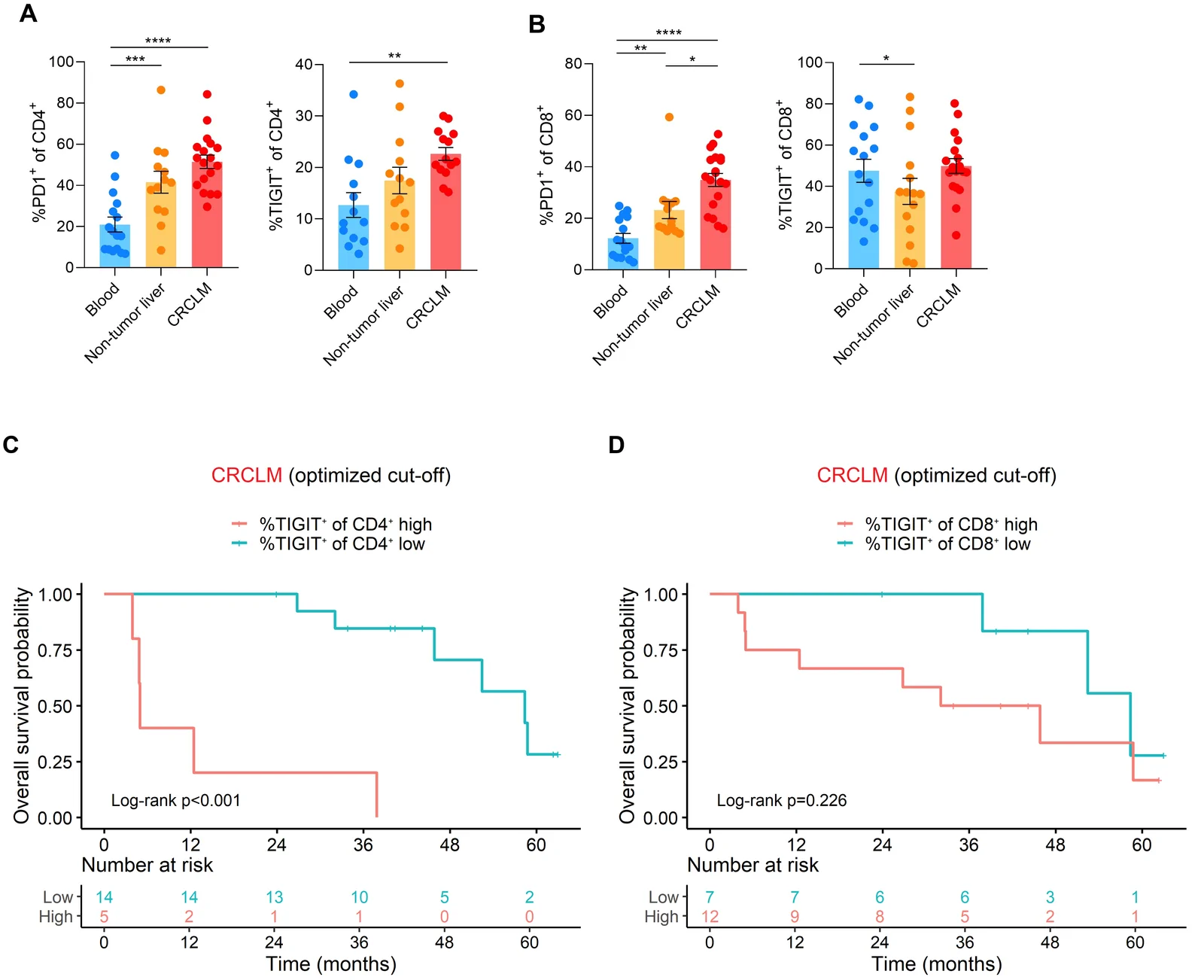
Checkpoint expression and TIGIT-associated survival in ex vivo expanded TILs from a separate cohort. (**A**,**B**) Quantitative plots showing the percentages of PD-1^+^ (**left** panel) and TIGIT^+^ (**right** panel). (**A**) CD4^+^ T cells and (**B**) CD8^+^ T cells from ex vivo expanded samples. (**C**,**D**) Kaplan–Meier survival curves comparing patients grouped by high versus low percentages of TIGIT-positive (**C**) CD4^+^ T cells and (**D**) CD8^+^ T cells from ex vivo expanded samples, stratified by the optimized cut-off value. Statistical analysis was performed using repeated-measures one-way ANOVA to compare the percentages of positive cells between different cell populations. *, *p* < 0.05, **, *p* < 0.01, ***, *p* < 0.001; ****, *p* < 0.0001. *p*-values from the log-rank test are indicated within the respective Kaplan–Meier plots. Kaplan–Meier panels show censor marks, log-rank *p* values, and numbers at risk; confidence bands are omitted for visual clarity. Survival analyses included 19 patients and 11 deaths. In panel C, the optimized groups comprised 14 low patients (6 deaths) and 5 high patients (5 deaths); HR for high versus low 17.47 (95% CI 3.23–94.59; log-rank *p* < 0.001). In panel (**D**), the optimized groups comprised 7 low patients (3 deaths) and 12 high patients (8 deaths); HR 2.24 (95% CI 0.59–8.56; log-rank *p* = 0.226).

Isolation of fresh TILs from resected tumor tissue requires substantial viable tumor material [19]. Ex vivo expansion from small tumor fragments offers a practical complementary strategy for analyzing tumor-resident T cells. To evaluate the applicability of this approach for immunophenotyping, we analyzed TILs from a separate patient cohort (n = 19) that underwent ex vivo IL-2-driven T cell expansion from CRCLM tissue. We compared cohort-level PD-1 and TIGIT patterns after ex vivo expansion with those observed in the separate fresh-tissue cohort. For PD-1, CD4^+^ T cells showed a significant increase in expression from blood to liver and from blood to tumor tissue (Figure 4A, left). Likewise, CD8^+^ T cells exhibited increased PD-1 expression from blood to liver and from liver to tumor (Figure 4B, left). For TIGIT, expanded CD4^+^ cells from non-tumor liver and CRCLM showed increased expression relative to blood, without a further increase between non-tumor liver and CRCLM (Figure 4A, right). In CD8^+^ T cells, TIGIT expression was significantly lower in liver tissue compared with blood, while no further change was observed in CRCLM tissue (Figure 4B, right). Together, the two independent cohorts provide complementary information on checkpoint expression patterns in freshly isolated and expanded T cells. We next explored survival in the expanded-cell cohort using the available follow-up. All 19 patients had usable survival data, with 11 deaths. At the optimized 28.7% cut-off, the groups comprised 14 low-expression patients (six deaths) and five high-expression patients (five deaths). High TIGIT^+^ CD4^+^ frequency was associated with shorter survival (HR 17.47, 95% CI 3.23–94.59; log-rank *p* < 0.001; cut-off-selection permutation *p* = 0.0010). With a median cut-off of 23.9%, the groups comprised 10 low; Figure 4C patients (four deaths) and nine high patients (seven deaths), and the association remained significant (HR 13.90, 95% CI 1.65–116.81; log-rank *p* = 0.0020). For TIGIT^+^ CD8^+^ cells, the optimized groups comprised seven low patients (three deaths) and 12 high patients (eight deaths); no significant association was observed (HR 2.24, 95% CI 0.59–8.56; log-rank *p* = 0.226). The median TIGIT^+^ CD8^+^ analysis was likewise negative (HR 0.98; Figure 4D, 95% CI 0.29–3.27; log-rank *p* = 0.975). No significant survival differences were detected for expanded non-tumor liver TIGIT^+^ CD4^+^ or CD8^+^ cells (median-cut-off log-rank *p* = 0.201 and *p* = 0.825, respectively). In a continuous Cox model, each 10-percentage-point increase in expanded CRCLM TIGIT^+^ CD4^+^ frequency corresponded to HR 2.68 (95% CI 0.96–7.51; *p* = 0.060). Diagnostics indicated that the association varied across expression levels and over follow-up (proportional hazards *p* < 0.001; quadratic-term *p* = 0.011), suggesting a more complex relationship than a uniform linear, time-constant effect. The concordant survival direction further supports TIGIT^+^ CD4^+^ cells as a candidate for investigation in expanded TIL models. These median-cut-off analyses are shown in Supplementary Figure S6.

Direct comparison of CRCLM samples across cohorts showed similar PD-1^+^ CD4^+^ frequencies (fresh: n = 16, median 55.55% [IQR 46.23–60.73]; expanded: n = 18, median 52.20% [40.95–58.50]; Holm-adjusted *p* = 0.617). TIGIT^+^ CD4^+^ frequencies were moderately higher in expanded samples (fresh: n = 16, 15.55% [11.69–23.23]; expanded: n = 14, 21.35% [19.83–26.25]), although this comparison did not remain significant after correction (adjusted *p* = 0.052). In the CD8^+^ compartment, expanded samples contained lower PD-1^+^ frequencies (fresh: n = 17, 48.70% [41.60–61.50]; expanded: n = 19, 36.20% [27.00–43.10]; adjusted *p* = 0.022) and higher TIGIT^+^ frequencies (fresh: n = 16, 16.85% [12.07–29.40]; expanded: n = 18, 48.30% [41.67–56.48]; adjusted *p* < 0.001; Supplementary Table S1). These unpaired comparisons demonstrate receptor- and subset-specific cohort differences rather than preservation of the native checkpoint phenotype.

### 3.5. Post Hoc Clinical Covariate Sensitivity Analyses

Clinical characteristics were balanced between the optimized TIGIT^+^ CD4^+^ low and high groups (Supplementary Table S2), with no nominal differences in either cohort (all *p* ≥ 0.058) for age, sex, primary tumor stage, systemic therapy before liver surgery, anti-VEGF or anti-EGFR therapy, synchronous presentation, number and bilobar distribution of metastases, or largest metastasis size. In Cox models adjusted for history of systemic therapy before liver surgery, the optimized high-versus-low association remained in the fresh cohort (adjusted HR 4.80, 95% CI 1.35–17.09; *p* = 0.015) and expanded cohort (adjusted HR 35.43, 95% CI 4.35–288.64; *p* < 0.001). Continuous and covariate analyses are reported in Supplementary Tables S2 and S3.

## 4. Discussion

This study identifies a distinct immune phenotype in CRCLMs characterized by enrichment of TIGIT-expressing CD4^+^ T cells that showed an exploratory association with shorter survival. CRCLMs show a shift in T cell composition compared to blood and adjacent non-tumor liver tissue, with increased CD4^+^ and reduced CD8^+^ T cells. In optimized cut-off analyses, elevated TIGIT expression on CD4^+^ tumor-infiltrating T cells—unlike PD-1 or TIGIT on CD8^+^ T cells—was associated with shorter overall survival. A TIGIT-high CD4 survival direction was also observed in a complementary cohort of ex vivo expanded TILs, supporting further investigation of this cell population. The reduced proportion of CD8^+^ T cells within CRCLM may be explained by several liver-specific immune mechanisms. The liver contains specialized antigen-presenting cells that can induce tolerance rather than productive T cell activation. Liver sinusoidal endothelial cells (LSECs), for example, are able to cross-present circulating antigens on MHC class I to naïve CD8^+^ T cells. Instead of generating full effector responses, this interaction can drive CD8^+^ T cells into a tolerized state through PD-L1-PD-1 signaling [22]. These findings suggest that tumor-derived antigens could preferentially dampen CD8^+^ T cell activity in the liver environment [23]. Another mechanism that may contribute to reduced CD8^+^ T cell numbers in liver metastases is the elimination of activated tumor-reactive T cells by hepatic myeloid cells. In a mouse model of CRCLMs, antigen-specific CD8^+^ T cells were deleted through Fas–FasL interactions with CD11b ^+^ F4/80^+^ macrophages in the metastatic liver tissue [5]. While our human data cannot directly demonstrate this mechanism, the relative depletion of CD8^+^ T cells within CRCLM is consistent with the idea that the hepatic metastatic niche can selectively impair CD8^+^ cytotoxic T cell responses. Recently, a study demonstrated that colorectal liver metastases suppress IL-18-driven γδ T cell activation and thereby reduce responsiveness to immune checkpoint blockade, further highlighting the profound immunoregulatory effects of the hepatic metastatic microenvironment [24]. Within this altered immune landscape, we observed a selective increase in TIGIT expression in CD4^+^ T cells inside CRCLM. TIGIT is an inhibitory receptor that binds the ligand CD155 and competes with the co-stimulatory receptor CD226 for the same ligand [25]. Previous studies in primary colorectal cancer reported that TIGIT expression is mainly associated with exhausted CD8^+^ T cells and correlates with poor prognosis [10]. Our results suggest that the situation in liver metastases is different. In CRCLM, TIGIT expression was predominantly observed on CD4^+^ T cells, whereas CD8^+^ T cells showed lower expression levels and no association with patient survival. Differences between primary tumors and metastatic lesions have been described in several studies. For example, comparisons of primary CRC and matched liver metastases revealed substantial differences in immune infiltration patterns and checkpoint receptor expression, including discordant PD-L1 expression between the two sites [26,27]. These findings support the idea that immune regulation in metastatic sites may differ significantly from that in the primary tumor. The identity of the TIGIT^+^ CD4^+^ T cell population in CRCLM remains an important question [28]. One possibility is that these cells represent Tregs. TIGIT is highly expressed on suppressive Tregs, and TIGIT signaling has been shown to stabilize their suppressive phenotype by inhibiting Th1-associated transcriptional programs such as T-bet and IFN-γ production [29]. In melanoma, tumor-infiltrating Tregs expressing high levels of TIGIT were associated with stronger immunosuppressive activity and poorer patient outcomes [30]. If the TIGIT-positive CD4 population observed in our CRCLM cohort represents Tregs, this could contribute to the association with reduced survival. However, alternative explanations are possible because conventional helper, cytotoxic, and dysfunctional CD4^+^ T cell states may also express TIGIT. The surface-marker panel underlying the present survival analysis did not distinguish regulatory from conventional or cytotoxic CD4^+^ T cells; TIGIT expression alone is therefore insufficient to assign lineage, function, or therapeutic vulnerability. Future studies should combine intracellular FOXP3 with CD25/CD127, cytotoxic effector molecules, functional assays, or single-cell transcriptomic analyses to define the responsible CD4^+^ population. An interesting observation in our dataset is that TIGIT expression on CD4^+^ T cells correlated only weakly with PD-1 expression. This suggests that TIGIT may mark a distinct regulatory pathway rather than simply reflecting classical PD-1-mediated exhaustion. Experimental work has shown that TIGIT and PD-1 can regulate T cell responses through partly independent signaling mechanisms, although both ultimately limit anti-tumor immunity [31]. In our cohort, PD-1 expression increased in the hepatic environment but did not predict survival, whereas TIGIT expression on CD4^+^ T cells did. This is consistent with, but does not establish, biologically distinct TIGIT-associated regulation within CRCLM.

The fresh and expanded analyses were performed in separate patient cohorts. A direct cross-cohort comparison showed similar PD-1^+^ CD4^+^ frequencies and a moderate increase in TIGIT^+^ CD4^+^ frequencies after expansion that did not remain significant after multiplicity correction. More pronounced differences occurred within the CD8^+^ compartment, with lower PD-1^+^ and higher TIGIT^+^ frequencies in expanded samples. Checkpoint-positive populations therefore remained detectable after expansion, but their proportions were not uniformly preserved. Because the cohorts were unpaired, the observed differences may reflect expansion conditions, cohort composition, or both. The expanded cohort is consequently best regarded as a complementary experimental model, while interpretation of the native CRCLM phenotype is based primarily on the freshly isolated samples.

This study should be interpreted in the context of its exploratory design. The primary fresh-tissue survival analysis included 16 evaluable patients with 12 deaths, and the expanded cohort included 19 patients with 11 deaths, resulting in wide confidence intervals around some effect estimates. Optimized cut-offs were selected in the same datasets and were therefore complemented by median-cut-off, permutation, multiplicity-adjusted, and continuous Cox analyses. The continuous estimates were directionally concordant; in the expanded cohort, model diagnostics suggested that the association may vary across expression levels and over follow-up rather than follow a uniform linear, time-constant pattern. Clinical comparisons and post hoc adjustment for preoperative systemic therapy supported the observed association, although the cohort size limited comprehensive multivariable modeling of treatment and metastatic burden. The fresh and expanded cohorts were independent, IL-2 expansion may influence checkpoint expression, and the CD4^+^ panel did not distinguish regulatory from conventional or cytotoxic CD4^+^ T cells. Accordingly, the survival association is best regarded as hypothesis-generating, while the concordant biological and survival patterns provide a strong rationale for prospective investigation.

## 5. Conclusions

In summary, TIGIT-expressing CD4^+^ T cells were enriched in colorectal cancer liver metastases, and higher frequencies were associated with shorter survival in exploratory dichotomized analyses. The continuous estimates were directionally similar. These findings highlight site-specific immune regulation in metastatic lesions and identify TIGIT^+^ CD4^+^ T cell enrichment as a promising candidate for prospective biomarker studies and mechanistic characterization.

## Figures and Tables

**Table 1:** Patient characteristics.

Variable		n = 36 n (%)
Age (years), median		63
Sex	Male	27 (75)
	Female	9 (25)
Body mass index (kg/m^2^), median		25.5
UICC stage	4A	28 (77.8)
	4B	3 (8.3)
	4C	5 (13.9)
T stage of primary CRC	1	1 (2.8)
	2	4 (11.1)
	3	28 (77.8)
	4	3 (8.3)
N stage of primary CRC	0	12 (33.3)
	1	14 (38.9)
	2	10 (27.8)
Metastasis	Synchronous	27 (75)
	Metachronous	9 (25)

## Data Availability

The data generated and analyzed during the study are available from the corresponding author upon reasonable request.

## Supplementary Materials

The following supporting information can be downloaded at: https://www.mdpi.com/article/10.3390/cancers18182977/s1. Figure S1: Representative flow-cytometry gating strategy used for freshly isolated and ex vivo expanded samples; Figure S2: CD4 expression is elevated within the CRCLM environment; Figure S3: PD-1 Expression Levels Are Not Associated with Survival in CRCLM Patients; Figure S4: TIGIT Expression Levels in Blood and Non-tumor Liver Are Not Associated with Survival; Figure S5: TIGIT Expression on CRCLM-Infiltrating CD4^+^ T-Cells is Independent of PD-1 Expression; Figure S6: Survival analyses of TIGIT expression in ex vivo expanded T cells using median cut-offs; Table S1: Comparison of PD-1 and TIGIT positivity in CRCLM T cell subsets from fresh and ex vivo expanded cohorts; Table S2: Clinical characteristics by optimized TIGIT^+^ CD4^+^ low and high groups; Table S3: Continuous Cox proportional-hazards models for checkpoint expression. Hazard ratios are reported per 10-percentage-point increase in marker-positive cells.
