# Supplementary material for: TIGIT+ CD4+ T Cell Enrichment in Colorectal Liver Metastases Is Associated with Shorter Survival
Source: Cancers (Basel). 2026 Sep 15;18(18):2977. doi: 10.3390/cancers18182977 (PMC13604289; doi:10.3390/cancers18182977)
Supplement: Supplementary file 1 [file cancers-18-02977-s001.zip › cancers-4474230-supplementary.pdf]

SUPPLEMENTARY FIGURES

Supplementary Figure S1

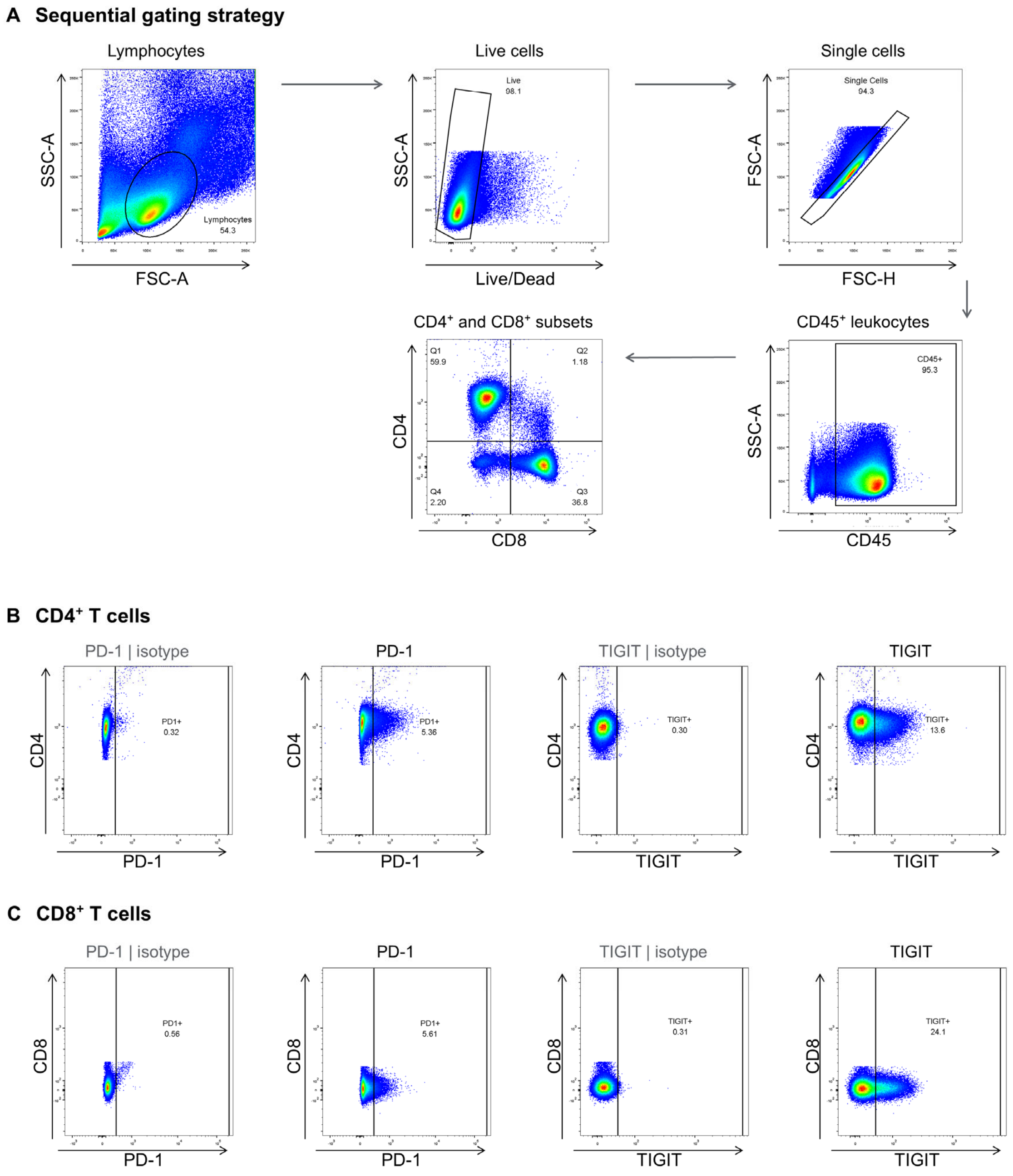

Supplementary Figure S1. Representative flow-cytometry gating strategy used for freshly isolated and ex vivo expanded samples. (A) Sequential gating of a representative peripheral-blood sample: lymphocytes were identified by FSC-A/SSC-A, followed by live-cell, singlet, and $CD45^+$ leukocyte gates. $CD4^+$ single-positive (Q1) and $CD8^+$ single-positive (Q3) subsets were selected; double-positive and double-negative events were excluded from subsequent analyses. (B, C) Representative PD-1 and TIGIT gates for $CD4^+$ (B) and $CD8^+$ (C) cells with the corresponding isotype-control plots. The same gating hierarchy was applied to freshly isolated and ex vivo expanded samples.

Supplementary Figure S2

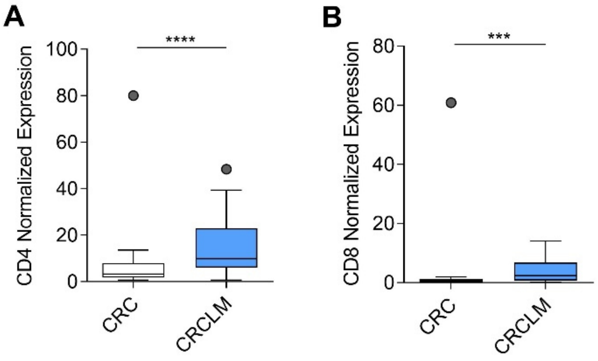

Supplementary Figure S2. CD4 expression is elevated within the CRCLM environment. (A, B) Comparison of (A) CD4 and (B) CD8 expression levels in matched primary CRC and CRCLM using bulk RNA sequencing data retrieved from the Gene Expression Omnibus (GEO) database (dataset: GSE207194).

Supplementary Figure S3

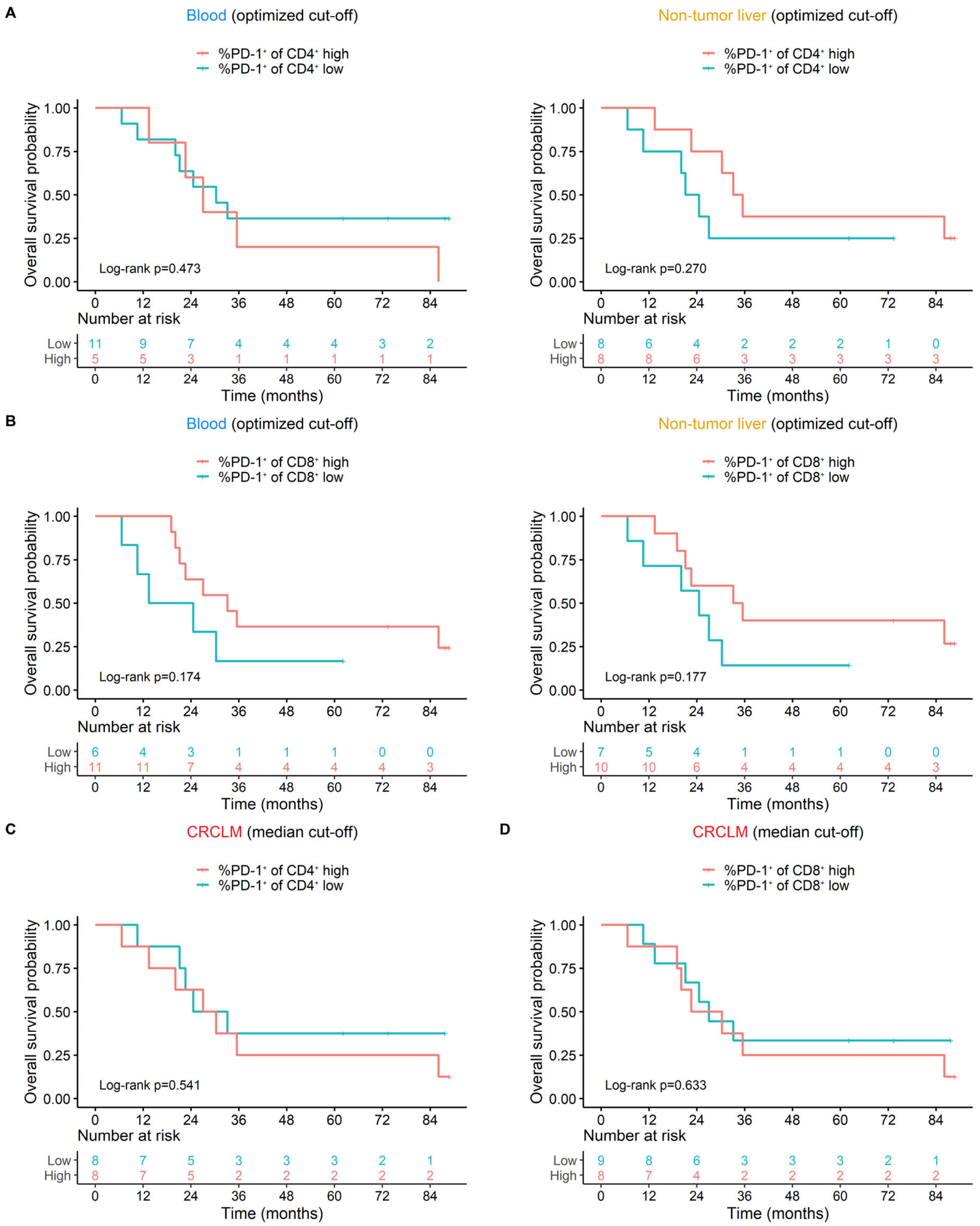

Supplementary Figure S3. PD-1 Expression Levels Are Not Associated with Survival in CRCLM Patients. (A) Kaplan-Meier survival curves comparing patients stratified into high and low PD-1 expression groups based on their expression on $CD4^{+}$ T cells in blood (left), and non-tumor liver tissue (right panel). (B) Kaplan-Meier survival curves comparing patients stratified into high and low PD-1 expression groups based on the expression on $CD8^{+}$ T cells in blood (left panel), and non-tumor liver tissue (right panel). (C-D) Kaplan-Meier survival curves based on median group separation in $CD4^{+}$ and $CD8^{+}$ T cells. P-values from the log-rank test are indicated within the respective Kaplan-Meier plots. Kaplan-Meier panels display censor marks, log-rank p-values, and numbers at risk. Confidence bands are omitted for visual clarity.

Supplementary Figure S4

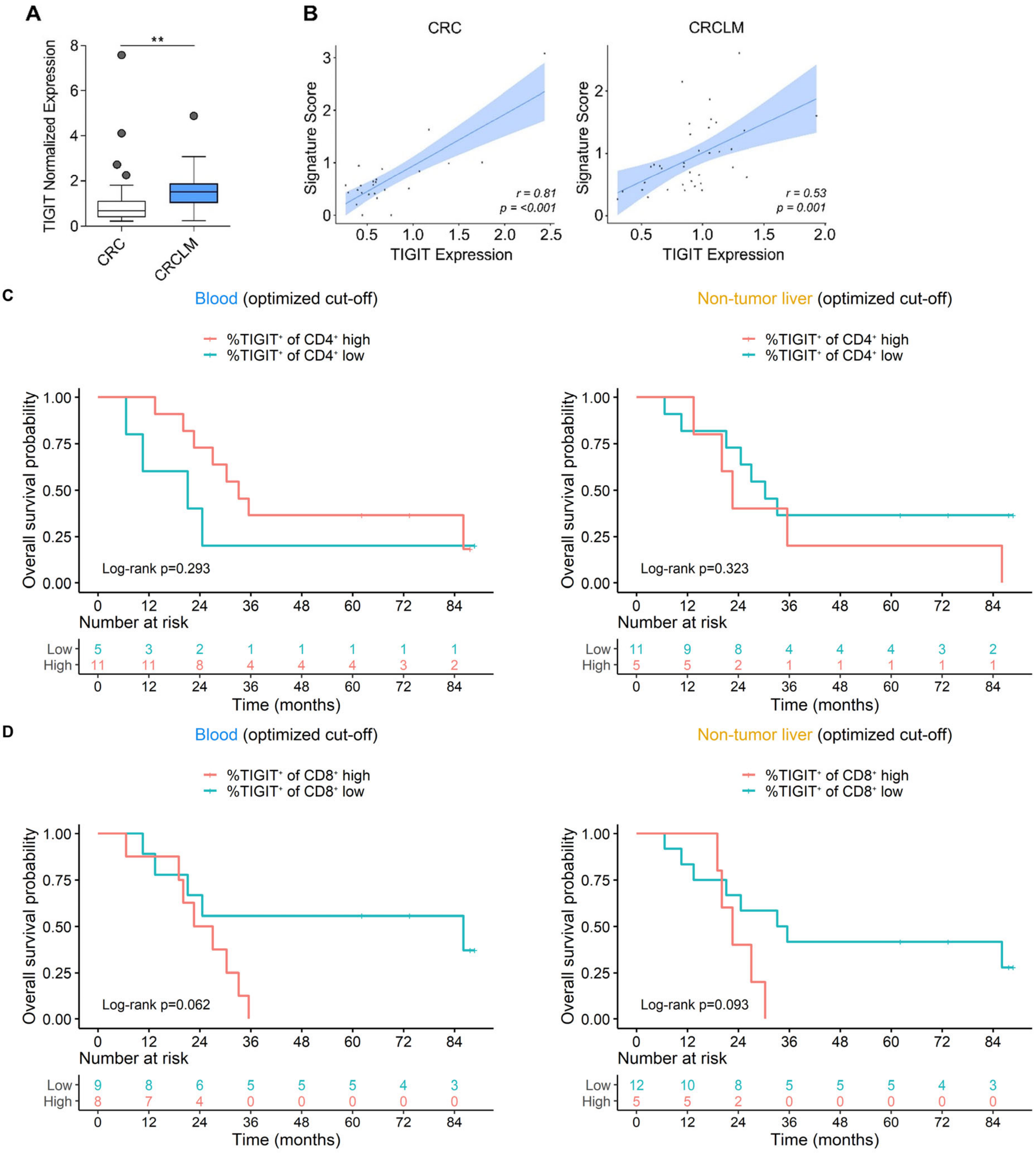

Supplementary Figure S4 (continued)

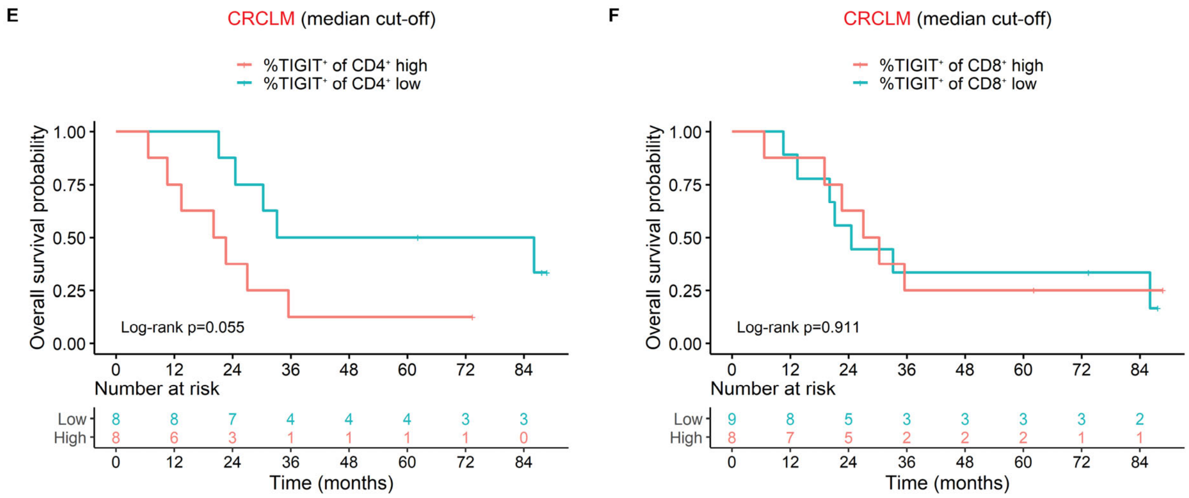

Supplementary Figure S4. TIGIT Expression Levels in Blood and Non-tumor Liver Are Not Associated with Survival. (A) Comparison of TIGIT expression levels in matched primary CRC and CRCLM using bulk RNA sequencing data retrieved from the Gene Expression Omnibus (GEO) database (dataset: GSE207194). (B) Analysis of the correlation between the exhausted T cell signature (comprised of the genes HAVCR2, LAG, PDCD1, CXCL13, LAYN) and TIGIT expression in both primary CRC (left) and corresponding CRCLM (right) within the same dataset. (C) Kaplan-Meier survival curves comparing patients stratified by high and low TIGIT expression on CD4$^+$ T cells blood (left panel), and non-tumor liver tissue (right panel). (D) Kaplan-Meier survival curves comparing patients stratified by high and low TIGIT expression on CD8$^+$ T cells in blood (left panel), and non-tumor liver tissue (right panel). (E-F) Kaplan-Meier survival curves based on TIGIT expression median group separation in CD4$^+$ and CD8$^+$ T cells. P-values from the log-rank test are indicated within the respective Kaplan-Meier plots. Kaplan-Meier panels display censor marks, log-rank p-values, and numbers at risk. Confidence bands are omitted for visual clarity.

Supplementary Figure S5

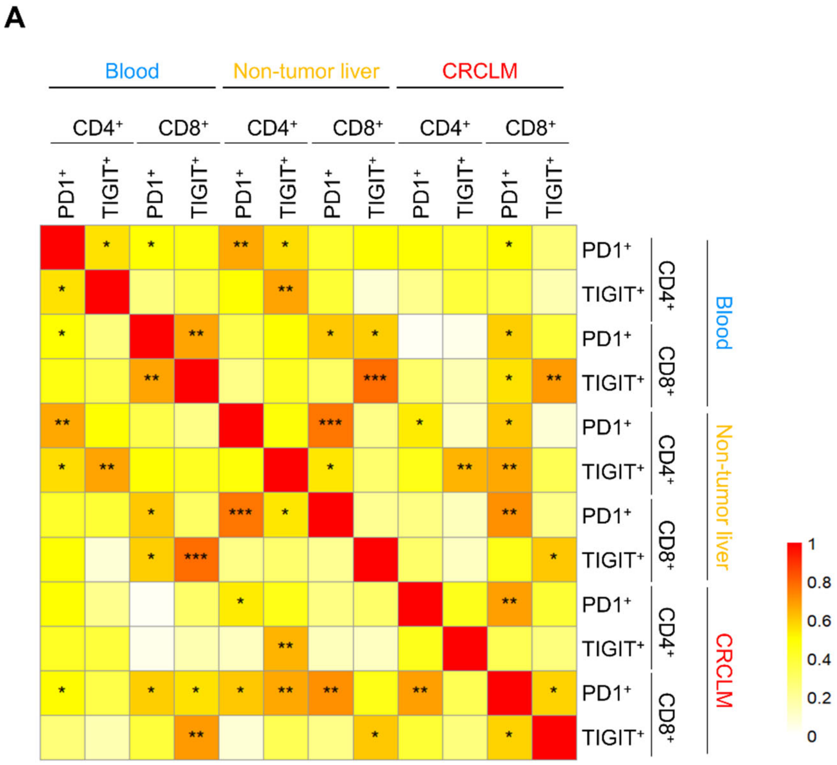

Supplementary Figure S5. TIGIT Expression on CRCLM-Infiltrating CD4^{+} T-Cells is Independent of PD-1 Expression. (A) Heatmap illustrating Pearson correlation coefficients for PD-1 and TIGIT expression within CD4^{+} and CD8^{+} T cell populations in peripheral blood, non-tumor liver tissue, and CRCLM. The presence of asterisks within marked rectangles indicates statistically significant correlations (*, $P < 0.05$, **, $P < 0.01$, ***, $P < 0.001$). The color gradient represents the strength and direction of correlation (R-value), with red corresponding to higher correlation levels.

Supplementary Figure S6

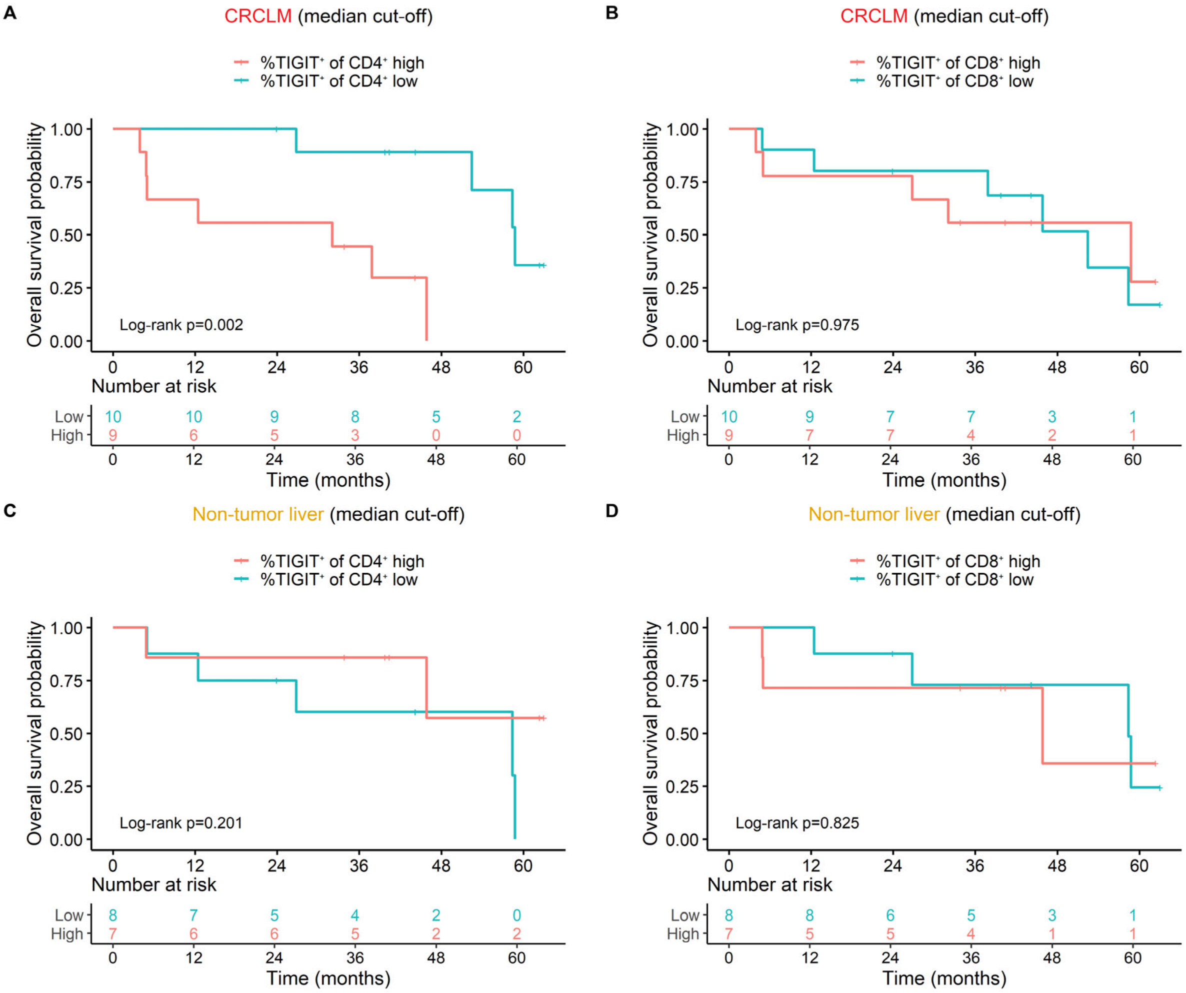

Supplementary Figure S6. Survival analyses of TIGIT expression in ex vivo expanded T cells using median cut-offs.

(A-B) Kaplan-Meier survival curves comparing patients stratified by median of high and low TIGIT expression on CRCLM (A) $CD4^+$ T cells, and (B) $CD8^+$ T cells. (C-D) Kaplan-Meier survival curves comparing patients stratified by median of high and low TIGIT expression in non-tumor liver tissue on (C) $CD4^+$ T cells, and (D) $CD8^+$ T cells. *P*-values from the log-rank test are indicated within the respective Kaplan-Meier plots. Kaplan-Meier panels display censor marks, log-rank p-values, and numbers at risk. Confidence bands are omitted for visual clarity.

SUPPLEMENTARY TABLES

Supplementary Table S1. Comparison of PD-1 and TIGIT positivity in CRCLM T-cell subsets from fresh and ex vivo expanded cohorts.

Marker/subset	Fresh cohort, n; median [IQR]	Expanded cohort, n; median [IQR]	U	Cliff's delta	p	Holm-adjusted p
PD-1$^+$ CD4$^+$	16; 55.55 [46.23-60.73]	18; 52.20 [40.95-58.50]	129.0	-0.104	0.617	0.617
TIGIT$^+$ CD4$^+$	16; 15.55 [11.69-23.23]	14; 21.35 [19.83-26.25]	166.0	0.482	0.026	0.052
PD-1$^+$ CD8$^+$	17; 48.70 [41.60-61.50]	19; 36.20 [27.00-43.10]	76.5	-0.526	0.007	0.022
TIGIT$^+$ CD8$^+$	16; 16.85 [12.07-29.40]	18; 48.30 [41.67-56.48]	274.5	0.906	<0.001	<0.001

Values are percentages of marker-positive cells. P values are from two-sided Mann-Whitney U tests; Holm-adjusted p values correct the four prespecified CRCLM marker/subset comparisons. Positive Cliff's delta values indicate higher frequencies in the expanded cohort.

Supplementary Table S2. Clinical characteristics by optimized TIGIT$^+$ CD4$^+$ low and high groups.

**Characteristic**	**Fresh low (n=10)**	**Fresh high (n=6)**	**p**	**Expanded low (n=14)**	**Expanded high (n=5)**	**p**
Age at liver surgery, years	61.5 [49.2-69.0]	59.0 [55.0-69.0]	0.957	62.0 [48.8-67.5]	65.0 [63.0-65.0]	0.486
Male sex	7/10 (70.0%)	5/6 (83.3%)	1.000	10/14 (71.4%)	4/5 (80.0%)	1.000
Rectal primary tumor	3/10 (30.0%)	3/6 (50.0%)	0.607	7/14 (50.0%)	4/5 (80.0%)	0.338
UICC stage IV at primary diagnosis	9/9 (100.0%)	3/4 (75.0%)	0.308	13/14 (92.9%)	3/5 (60.0%)	0.155
Primary tumor T stage 3-4	8/10 (80.0%)	5/6 (83.3%)	1.000	13/14 (92.9%)	5/5 (100.0%)	1.000
Node-positive primary tumor	8/10 (80.0%)	5/6 (83.3%)	1.000	7/14 (50.0%)	3/5 (60.0%)	1.000
History of systemic therapy before liver surgery	10/10 (100.0%)	5/6 (83.3%)	0.375	12/14 (85.7%)	3/5 (60.0%)	0.272
History of anti-VEGF therapy	4/10 (40.0%)	1/6 (16.7%)	0.588	0/14 (0.0%)	2/5 (40.0%)	0.058
History of anti-EGFR therapy	4/10 (40.0%)	4/6 (66.7%)	0.608	3/14 (21.4%)	1/5 (20.0%)	1.000
Synchronous liver metastases	7/10 (70.0%)	4/6 (66.7%)	1.000	12/14 (85.7%)	3/5 (60.0%)	0.272
Multiple liver metastases	9/10 (90.0%)	5/6 (83.3%)	1.000	10/14 (71.4%)	4/5 (80.0%)	1.000
Bilobar liver metastases	7/10 (70.0%)	4/6 (66.7%)	1.000	10/14 (71.4%)	3/5 (60.0%)	1.000
Largest liver metastasis >5 cm	0/8 (0.0%)	1/4 (25.0%)	0.333	3/14 (21.4%)	1/5 (20.0%)	1.000

Continuous values are median [IQR]; categorical values are n/N (%). P values are from Wilcoxon rank-sum tests or Fisher exact tests.

Supplementary Table S3. Continuous Cox proportional-hazards models for checkpoint expression. Hazard ratios are reported per 10-percentage-point increase in marker-positive cells.

Cohort	Compartment	Marker/subset	N/events	HR (95% CI)	p	PH p	Quadratic p
Fresh	Blood	PD-1 / CD4$^+$	16/12	1.13 (0.40-3.17)	0.813	0.076	0.063
Fresh	Blood	PD-1 / CD8$^+$	17/13	1.11 (0.74-1.65)	0.617	0.112	0.450
Fresh	Blood	TIGIT / CD4$^+$	16/12	0.48 (0.17-1.36)	0.168	0.080	0.491
Fresh	Blood	TIGIT / CD8$^+$	17/13	1.18 (0.85-1.65)	0.327	0.024	0.338
Fresh	Non-tumor liver	PD-1 / CD4$^+$	16/12	0.85 (0.51-1.43)	0.551	0.039	0.126
Fresh	Non-tumor liver	PD-1 / CD8$^+$	17/13	0.89 (0.68-1.15)	0.360	0.401	0.954
Fresh	Non-tumor liver	TIGIT / CD4$^+$	16/12	1.40 (0.34-5.72)	0.640	0.452	0.334
Fresh	Non-tumor liver	TIGIT / CD8$^+$	17/13	1.55 (0.88-2.74)	0.129	0.050	0.267
Fresh	CRCLM	PD-1 / CD4$^+$	16/12	1.33 (0.77-2.32)	0.308	0.944	0.441
Fresh	CRCLM	PD-1 / CD8$^+$	17/13	1.04 (0.77-1.42)	0.792	0.678	0.377
Fresh	CRCLM	TIGIT / CD4$^+$	16/12	1.69 (0.82-3.45)	0.153	0.538	0.677
Fresh	CRCLM	TIGIT / CD8$^+$	17/13	1.04 (0.63-1.71)	0.885	0.750	0.812
Expanded	Blood	PD-1 / CD4$^+$	16/8	1.51 (0.96-2.40)	0.077	0.161	0.313
Expanded	Blood	PD-1 / CD8$^+$	16/8	1.41 (0.53-3.75)	0.493	0.179	0.918
Expanded	Blood	TIGIT / CD4$^+$	16/8	1.60 (0.77-3.33)	0.205	0.017	0.179
Expanded	Blood	TIGIT / CD8$^+$	16/8	1.15 (0.78-1.69)	0.478	0.125	0.105
Expanded	Non-tumor liver	PD-1 / CD4$^+$	15/7	0.93 (0.64-1.35)	0.708	0.577	0.193
Expanded	Non-tumor liver	PD-1 / CD8$^+$	15/7	1.06 (0.60-1.86)	0.851	0.328	0.344
Expanded	Non-tumor liver	TIGIT / CD4$^+$	15/7	1.08 (0.56-2.11)	0.811	0.043	0.254
Expanded	Non-tumor liver	TIGIT / CD8$^+$	15/7	1.12 (0.77-1.62)	0.555	0.014	0.146
Expanded	CRCLM	PD-1 / CD4$^+$	19/11	1.20 (0.84-1.72)	0.307	0.757	0.688
Expanded	CRCLM	PD-1 / CD8$^+$	19/11	0.97 (0.56-1.68)	0.915	0.599	0.315
Expanded	CRCLM	TIGIT / CD4$^+$	19/11	2.68 (0.96-7.51)	0.060	<0.001	0.011
Expanded	CRCLM	TIGIT / CD8$^+$	19/11	0.98 (0.75-1.30)	0.912	0.182	0.290

CI, confidence interval; HR, hazard ratio; PH p, p value from the scaled Schoenfeld-residual test; Quadratic p, p value for a quadratic term testing departure from log-linearity. All p values are two-sided.
